# The Dietetics of the Channel Swim

**Published:** 1911-09-23

**Authors:** J. W. Burgess

**Affiliations:** Lorne Cottage, 30 Dover Street, Walmer.


*To the Editor of* The Hospital.

### THE DIETETICS OF THE CHANNEL SWIM.

*To the Editor of* The Hospital.

Sir,—It having been stated and advertised in the public press that during my Channel swim I partook of various forms of nourishment, I think it only fair to state publicly that my only food consisted of Swiss milk chocolate and a few grapes and the leg of a chicken.—Yours faithfully, J. W. Burgess.

Lorne Cottage, 30 Dover Street, Walmer.

[We understand that Mr. Burgess used Nestlé's milk chocolate, and also consumed half a cup of tea made with Nestlé's Swiss milk. No meat extracts were used by him. —Ed. The Hospital.]

